# AKT-ions with a TWIST between EMT and MET

**DOI:** 10.18632/oncotarget.11232

**Published:** 2016-08-11

**Authors:** Huifang Tang, Daniela Massi, Brian A. Hemmings, Mario Mandalà, Zhengqiang Hu, Andreas Wicki, Gongda Xue

**Affiliations:** ^1^ Department of Pharmacology, Zhejiang University, School of Basic Medical Sciences, Hangzhou, China; ^2^ Department of Surgery and Translational Medicine, University of Florence, Florence, Italy; ^3^ Department of Mechanisms of Cancer, Friedrich Miescher Institute for Biomedical Research, Basel, Switzerland; ^4^ Department of Oncology and Hematology, Papa Giovanni XXIII Hospital, Bergamo, Italy; ^5^ Department of Biomedicine, University Hospital Basel, Basel, Switzerland

**Keywords:** epithelial-mesenchymal transition, Twist, Akt, plasticity, phosphorylation

## Abstract

The transcription factor Twist is an important regulator of cranial suture during embryogenesis. Closure of the neural tube is achieved via Twist-triggered cellular transition from an epithelial to mesenchymal phenotype, a process known as epithelial-mesenchymal transition (EMT), characterized by a remarkable increase in cell motility. In the absence of Twist activity, EMT and associated phenotypic changes in cell morphology and motility can also be induced, albeit moderately, by other transcription factor families, including Snail and Zeb. Aberrant EMT triggered by Twist in human mammary tumour cells was first reported to drive metastasis to the lung in a metastatic breast cancer model. Subsequent analysis of many types of carcinoma demonstrated overexpression of these unique EMT transcription factors, which statistically correlated with worse outcome, indicating their potential as biomarkers in the clinic. However, the mechanisms underlying their activation remain unclear. Interestingly, increasing evidence indicates they are selectively activated by distinct intracellular kinases, thereby acting as downstream effectors facilitating transduction of cytoplasmic signals into nucleus and reprogramming EMT and mesenchymal-epithelial transition (MET) transcription to control cell plasticity. Understanding these relationships and emerging data indicating differential phosphorylation of Twist leads to complex and even paradoxical functionalities, will be vital to unlocking their potential in clinical settings.

## INTRODUCTION

## EMT AND SIGNALING

**Figure 1:**
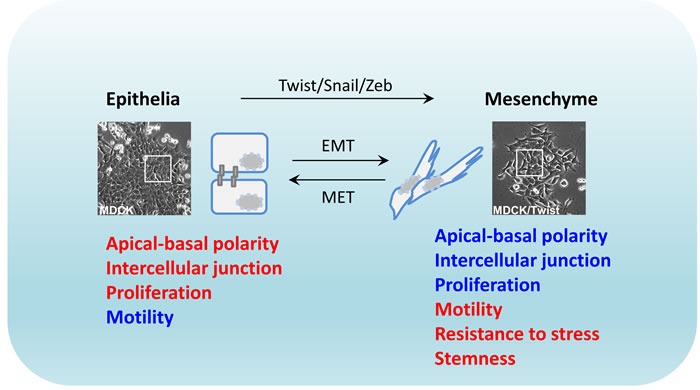
Plastic epithelial-to-mesenchymal transition Transcriptional regulation of EMT by activated Twist, Snail and Zeb oncogenic proteins is often accompanied with cellular morphological change. EMT: epithelial-mesenchymal transition; MET: mesenchymal-epithelial transition. Red: high level; blue: low level.

EMT is an ancient developmental process that is characterized by morphological changes in epithelial cells, whereby they acquire a mesenchyme phenotype [1]. The hallmarks of EMT are functionally decreased adhesive capacity and increased mobility [2]. This is facilitated by epithelial phenotype changes where cells switch from apical-basal polarity to anterior-posterior polarity, characteristically illustrated with disrupted intercellular junctions and increased migratory potential of individual cells. The basis of EMT in physiology is to remove/replace unnecessary epithelia at specific locations or developmental stages. Biochemically, EMT cells exhibit distinct patterns of upregulated gene expression of proteins engaged in remodeling of cell-cell contact, cytoskeleton, and interaction with extracellular matrix (ECM) [3]. In the last two decades, a group of transcription factors including the Twist, Snail and Zeb families, have been identified as the EMT-inducing transcription factors governing the EMT process *in vitro*, as inducers, enhancers, or both [4]. Overexpression of these proteins in untransformed mammalian epithelial cells induces potent cell scattering phenotypes that also functionally resemble the original EMT in mouse model [5, 6]. Therefore, the EMT process is generally recognized as a reprogramming event essentially driven at the transcriptional level, whilst being initiated and coupled with ECM signaling [7, 8] (Figure 1).

Many pathways are associated with EMT. One of the well-documented signaling axes is the transforming growth factor (TGF) cascade [9] that induces cell scattering with increased invasive potential in a variety of cancer cells with epithelial origin [10, 11]. In addition to TGFβ, the receptor tyrosine kinases (RTKs) such as EGFR family [12], c-Met [13], VEGFR [14, 15], PDGFR [16] and others (such as Wnt pathway [17, 18]) are all capable of initiating and/or maintaining EMT phenotypes. Importantly, activation of these membrane-associated signaling complexes often correlates with elevated expression level of the EMT-inducing transcription factors, and conversely, in addition to maintaining the EMT gene signature, can also maintain and enhance RTK signaling through feedback or feed-forward signaling loops [19, 20]. With a particular focus on understanding these mechanisms connecting the EMT transcriptional response with the activation of the upstream signalosomes, a number of studies revealed that three intracellular cascades, mTOR/PI3K/Akt, MAPK and Rho GTPases are core mediators transducing signals to activate the EMT-inducing transcription factors [21–24]. These findings have been demonstrated in many types of carcinomas, with great consistency [25].

The invasive behaviors of metastatic cancer cells closely resemble the physiological EMT phenotypes and effectively respond to the known upstream signaling cascades. Metastasis initiates with a small portion of the cancer cells within the primary organ disseminating from the tumor mass and by expressing remarkably high level of proteases, digest matrix barriers and subsequently invade into surrounding tissues [26, 27]. Local invasive cancer cells further intravasate into lymphatic and/or blood vessels and transit to distant organs where they extravasate and re-colonize as a metastatic tumour lesion [28, 29]. A specific biological impact of the EMT inducers in cancer metastasis is demonstrated by depletion of the EMT inducers in invasive cancer cells, which significantly attenuates the metastatic spread, whilst in most cases having little to no effect on the primary tumor growth [30]. This indicates that cancer cells preferentially take advantage of the physiological EMT signaling to support their invasion.

## THE PLASTICITY OF EMT

Although TGF signaling triggers robust EMT activation in epithelial cells, a reversal of the EMT phenotype was observed when TGFβ signaling was disrupted by withdrawal [31] or pharmacological inhibition [32], indicating a high degree of plasticity in the EMT process [33]. In an *in vivo* orthotopic metastatic mouse model, such reversal was discovered in the cancer cells that had undergone EMT to colonize and establish the metastatic tumour. It was found that the activity of Twist was significantly reduced at the initial phase of metastatic colonization in the lung and the cancer cells re-exhibited a classical epithelial phenotype with decreased migratory potential, with this process being coined MET (mesenchymal-to-epithelial transition) [34]. In contrast to the EMT phenotype which supports the cancer cell invasion but restricts their proliferation, MET favors re-activation of the proliferative potential, but limits invasiveness. In addition to Twist, other EMT inducers also displayed downregulated activity during the establishment of metastatic growth [35], and MET was postulated to be essential to maintain cancer cell survival in metastatic sites [36, 37]. Moreover, experimental evidence also demonstrated that the cells with phenotypic characters of EMT antagonize stress-induced apoptosis, including resistance to hypoxic pressure and DNA-damaging reagents. These observations support a hypothesis that the cancer cells with EMT properties potentially possess a “stemness” capacity [38–40], although many aspects of this concept are still to be convincingly demonstrated [41].

## REGULATION OF THE EMT-INDUCING TRANSCRIPTION FACTOR TWIST

Considering the interplay between membrane-associated signaling complexes and EMT-inducing transcription factors, as well as the impact of EMT on cancer metastasis, it is essential to understand the fundamental mechanisms of how signals are transduced to activate the EMT inducers such as Twist. Twist belongs to the basic helix-loop-helix (bHLH) transcription factor family. In mammals, Twist exists as two forms that are crucial for proper prenatal development [42, 43], although their functional roles in postnatal physiology are still vague. Across species from fruit fly to human, it is evolutionarily conserved and recognizes a palindromic-like consensus sequence CANNTG, also called E-box, in the proximal region of promoters. Its binding capacity is preferentially mediated through hetero-dimerization of Twist with other family members to implement its transcriptional regulation [44]. A recent study employing ChIP coupled with high-throughput sequencing for the analysis of Twist-binding DNA elements revealed that Twist can bind to two tandem E-boxes, a unique feature that distinguishes it from other bHLH transcription factors [45]. To date, clinical studies have shown evidence to support a pro-metastatic role for Twist regulated EMT gene expression during cancer progression [46–49]. This includes a number of studies indicating that overexpression of Twist enhances carcinoma metastasis [50, 51] and associates with unfavorable clinical prognosis [52–55]. Mechanistically, Twist-promoted cancer metastasis is mediated through its transcription activity that is hijacked by cancer cells [25]. Twist binding to E-boxes can transcriptionally repress E-cadherin expression, and consequently disrupts the intercellular adhesion and induce single cancer cell dissemination from the primary location [56]. In parallel, Twist overexpression remodels cytoskeleton and upregulates several essential signaling molecules such as Akt2 and TGFβ2 to robustly induce the EMT phenotype [57].

### Differential phosphorylation of Twist as a functional switch

**Figure 2:**
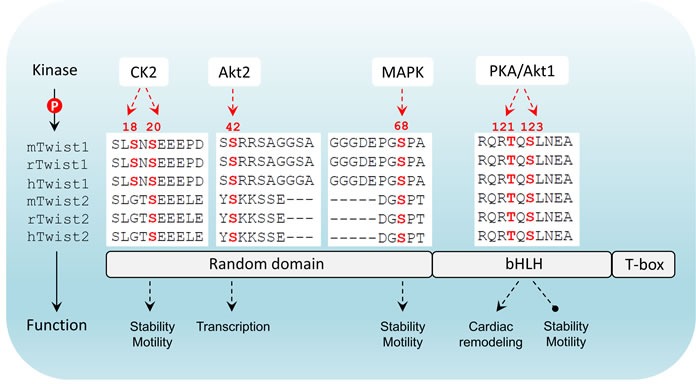
Twist structure and phosphorylation conservation in mammals Twist has three major domains including a N-terminal flexible domain, a basic helix-loop-helix domain that is responsible for DNA-binding and a C-terminal Twist-box. Differential phosphorylation patterns that have been reported are indicated. In mammals there are two members in Twist family. The phosphorylated amino acids highlighted in red are highly conserved in both members across three species (m: mouse; r: rat; h: human). CK2: casein kinase 2; bHLH: basic helix-loop-helix; T-box: Twist-box.

**Figure 3:**
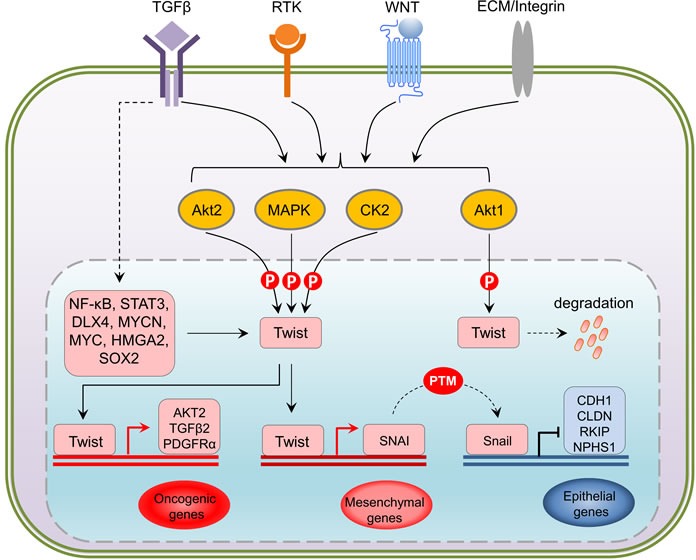
Upstream and downstream regulation of Twist At transcriptional level, NF-κB, STAT3, DLX4, MYCN, MYC, HMGA2 and SOX2 have been shown to upregulate Twist in response to the activation of TGFbeta, RTKs, WNT and Integrin pathways. Being a central hub, translated Twist undergoes differential phosphorylation directly mediated by Akt, MAPK and CK2 kinases in a context-dependent manner. Activation of Twist via phosphorylation triggers oncogenic gene expression such as AKT, TGFB and PDGFR, and represses E-cadherin (CDH1), Claudins (CLDN), Raf kinase inhibitor protein (RKIP) and Nephrin1 (NPHS1) that are crucial for cell-cell contact through its downstream target Snail, an epigenetic event that actively leads to an reinfoced EMT activation. PTM: post-translational modification.

Interestingly, TGFβ2 upregulation by Twist can be enhanced by Akt-mediated phosphorylation of Twist on serine 42 (S42), which increases binding to the TGFβ2 promoter. Thus, Akt-directed Twist phosphorylation on S42 is crucial for the crosstalk between PI3K/Akt and TGFβ pathways in metastatic breast cancer [57] and is also demonstrated to be an invasive signature in other cancer models [58]. Not limited to S42 phosphorylation, independent studies have highlighted other phosphorylation sites on Twist including serine 68 (S68) [59], serine 18 (S18) and serine 20 (S20) [60], as well as threonine 121 (T121) and serine 123 (S123) [61] that are differentially phosphorylated by MAPK (JNK, ERK, p38), casein kinase 2 and Akt, respectively, most of which are suggested to activate and/or enhance Twist functions through promoting its stability in a context-dependent manner. Analysis of the protein sequences of Twist family members in mammals demonstrates that all these crucial phosphorylation sites are evolutionarily conserved (Figure 2), implying that the Twist proteins across species potentially share a functional homology and the two Twist isoforms may display redundant functions. Clearly, Twist-phosphorylating kinases are mainly the two important intracellular signaling mediators Akt and MAPK, both of which are indisputably involved in cell proliferation, differentiation and invasion in cancer cells and key players of drug resistance in clinic. These two nodes can be activated by drivers of EMT and metastasis including well-known receptor signaling kinases such as TGFβ, RTKs, ECM-mediated integrin pathway and canonical or non-canonical WNT signaling, which in turn facilitate EMT and cancer metastasis *via* regulation of Akt and MAPK. The increasing evidence demonstrating the activity of Twist in regulation of cell migration and invasion which are controlled by a posttranslational modifications may have many critical implications for controlling metastatic lesions, when cancer cells need to re-establish high levels of proliferation and growth. Intriguingly, the basal level of Twist in non-neoplastic or non-metastatic cells is generally low, indicating that Twist activation starts from an activated transcription program. To date, it has been shown that dependent upon the types of malignancy, Twist can be transcriptionally upregulated by NF-κB [62, 63], STAT3 [64–66], DLX4 [67], MYCN and MYC [68], HMGA2 [69] and SOX2 [70],, indicating that activation of Twist is a coordinated event between epigenetic transcriptional regulation and post-translational modification. Following phospho-activation, as a core element involved in the activation of transcriptional complexes, Twist can drive the transcription of a number of target genes, many of which are oncogenic. Although Twist was shown to be a transcriptional repressor that inversely correlates with E-cadherin expression [71], it may possibly not be directly involved in suppressing CDH1 transcription, rather, this is suggested to be mediated by Snail2, a direct transcription target downstream of Twist [72]. It should also be noted that the activation of Snail proteins, like Twist, is also under the control of phosphorylation [73]. Depending on the upstream kinases, phosphorylated Snail may exhibit enhanced repressing activity [74], or rapid degradation [75]. Direct binding of Snail proteins within the regulatory region of CDH1 has been reported to mediate its repressing [76] (Figure 3). Thus, it seems that the repression of CDH1 transcription is a sequential program controlled by phospho-activated Twist/Snail axis. A recent study focusing on the specificity of Twist-mediated transcription, revealed that Twist dimers can uniquely recognized a tandem stretch of E-boxes [45]. The biological consequence of such interaction in relation to preferential to transcriptional activation, repression, or both, remains to be determined. Undoubtedly, whether any related transcriptional specificity is correlated with the multifaceted phosphorylation patterns is also an essential question.

### Inhibition and reversal of Twist-induced EMT at the site of metastasis

The metastasizing cancer cells displaying an EMT phenotype are found to exhibit higher resistance to environmental stress [77, 78]. High expression level of EMT signatures observed in circulating invasive cells supports survival in the blood stream [79, 80], allowing subsequent extravasation of these cells from the blood or lymphatic vessels and establishment in distant organs/tissues. Suppression of the EMT capability in the circulating cancer cells can efficiently attenuate the anti-apoptotic competence [81]. Therefore, the EMT state actively supports cancer cells to overcome environmental stress. As mentioned above, the signaling mediators that link extracellular signals to EMT inducers often converge to two major signaling nodes PI3K/Akt and MAPK, both of which are responsible for drug resistance to clinical therapies in many types of cancer [82]. Indeed, the metastatic cancer cells associated with EMT clearly show remarkable resistance to a number of small molecular inhibitors in clinic [83–85]. As EMT programming limits cell proliferation, it is critical for re-establishing cancer cells at the distant metastatic site to overcome this barrier for colonization. In this regard, it raises the fundamental question of how EMT is revered, or more specifically how EMT inducers, such as Twist, are deactivated. Whilst further insights are being made, the current understanding remains somewhat limited, with changes in Twist stability the best described. In different types of cancer cells, Twist instability can be induced by changes in microRNA [86–88]. In addition to the regulation of Twist stability at RNA level, two notable studies suggest that deactivation of Twist in metastatic lesion may be triggered by elevated protein instability, mediated by either de-phosphorylation of S68 by the small C-terminal Domain Phosphatase 1 [89], or Akt1-directed phosphorylation of Twist on T121 and S123 that promotes β-TrCP-mediated Twist1 ubiquitination and degradation [90]. These two discoveries elucidate the suppression of Twist1 activity through potential kinase-regulated autonomous feedback signaling and further highlights the developing contribution of phosphorylation to the regulation of the EMT process.

## DISCUSSION

Metastatic spread is a key issue in clinical cancer therapy because it represents the major cause of death for cancer patients. Many types of invasive cancer with epithelial origin represent an advanced EMT phenotype that exhibits a high grade of plasticity. Establishment and outgrowth of metastases in distant organs primarily requires local re-colonization, which is reflected by decreased motility and increased proliferation potential of the cancer cells. Such reversal from EMT to MET in breast cancer-associated metastases was experimentally recognized in Twist-induced metastatic models [34, 35]. However, the mechanisms of how Twist activity is regulated during the establishment of metastases is currently undetermined. What factors in the metastatic site determine the need of its inactivation? Recent studies in mouse model shed some lights on these issues [90]. Cancer cell proliferation in the metastatic site requires high level of global Akt activity which is principally capable of inducing cell migration, and Twist was reported to inhibit environmental stress-triggered senescence [91, 92]. The opposing roles of Akt1 and Akt2 during metastatic progression [93, 94] may provide a ration interpretation by proposing a model whereby when cancer cells need to disseminate, Akt2-mediated phosphorylation on S42 of Twist is dominant; whilst during metastatic colonization, Akt1-mediated phosphorylation on T121 and S123 triggers ubiquitination-dependent degradation of Twist and promotes proliferation. This would imply the existence of a dynamic pool of Akt isoforms that differentially regulates cancer cell behavior at both primary and metastatic sites. If this is true *in vivo*, the predominance of individual Akt isoforms will be the fundamental factor to determining the cancer cell behavior and fate. Interestingly, independent study has shown that Akt isoforms are individually regulated by specific microRNAs thus distinguishes the spatiotemporal Akt isoforms-contributed activity [95].

This model whereby Twists oncogenic functions are regulated by the predominant Akt isoforms raises further crucial questions to be elucidated : 1) what are the signals in cancer cells that would control the switch of Twist-phosphorylating modulators between the Akt1 and Akt2 isoforms? 2) How do Akt1 and Akt2 compete with each other to impose dominant control of Twist phosphorylation? 3) What controls the Akt1 and Akt2 ratio specifically in the metastatic lesion? These questions still remain unanswered and studies have reported contradictory observations, including a structural study of Twist linking T121 and S123 phosphorylation in prostate cancer to Twist mediated metastasis, as opposed to Twist degradation [96]. This discrepancy may indicate difference between cancer types or progression stages. An explanation for the dominating status of Akt1 and Akt2 may be that there exists a feed-back signaling loop through multiple signaling cross-talks that eventually leads to microRNA-mediated degradation of individual Akt isoforms, thereby modulating entire Akt pool activity [95, 97]. Alternatively, it is also proposed that distinct phosphorylation patterns of Twist require different co-activators, which may determine the selectivity of phosphorylation motifs on Twist exposed to Akt isoforms. In fact, such phospho-specific phenotypes coupled with distinct cell fate in cancer have been also found similar to other EMT-drivers such as Snail family [75, 98]. Thus, phosphorylation of EMT-promoting molecules seems to, at least in part, elaborately act as a functional “on/off” signal in favor of cancer cell migration, invasion and survival. Moreover, on-site de-phosphorylation by specific phosphatases could also contribute, either directly through targeting Twist, or indirectly by deactivating upstream kinases such as tyrosine receptors, to influence Twist stability. Insights into this may be found in the activity of SHP2 in a well-studied metastasis model, which is tremendously increased and promotes metastatic colonization [99].

Additionally, more experimental data is needed to explore the mechanistic interplay between Twist (and/or others)-induced EMT and metastasis. A recent study challenged the conventional role of Twist-induced EMT in promotion of metastasis. In a pancreatic cancer model, depletion of Twist or Snail did not inhibit pancreatic metastasis; rather, EMT was crucial for the tumors to resist to the treatment of DNA-damaging reagents [100], another important notion closely related to current clinical cancer therapies [101, 102]. Moreover, it would be interesting to know whether Twist phosphorylation on different sites occurs simultaneously, individually or step-wisely. Does one site phosphorylation/de-phosphorylation influence the others? The answers to these questions will facilitate a better understanding of the affiliated signaling events that regulate the transition between EMT and MET during cancer progression, which will greatly facilitate development of diagnostic tools for clinical applications. Furthermore, such phospho-pattern-specific biomarkers may not only predict whether the cancer cells in metastatic tumors start a second wave of dissemination, but also be considered as potential druggable targets [103].
